# Effects of Freeze–Thaw Cycles on Strength and Wave Velocity of Lime-Stabilized Basalt Fiber-Reinforced Loess

**DOI:** 10.3390/polym14071465

**Published:** 2022-04-04

**Authors:** Wensong Wang, Guansen Cao, Ye Li, Yuxi Zhou, Ting Lu, Binbin Zheng, Weile Geng

**Affiliations:** 1 State Key Laboratory of Geohazard Prevention and Geoenvironment Protection, Chengdu University of Technology, Chengdu 610059, China; wws@cdut.edu.cn (W.W.); liye_054008@163.com (Y.L.); zyx@cdut.edu.cn (Y.Z.); 2 State Key Laboratory of Coal Mine Disaster Dynamics and Control, Chongqing University, Chongqing 400044, China; tlu@cqu.edu.cn (T.L.); gwle@cqu.edu.cn (W.G.); 3 Zijin Mining Company Limited, Shanghang 364200, China; 4 School of Management Science and Engineering, Shandong Technology and Business University, Yantai 264005, China; zhengbin_vip@sina.com

**Keywords:** fiber-reinforced loess, freeze–thaw cycles, lime stabilization, unconfined compressive strength, wave velocity

## Abstract

Basalt fiber is a new environmentally-friendly material with excellent potential for soil reinforcement in geotechnical engineering construction. This study explores the effects of freeze–thaw cycles on the unconfined compressive strength (UCS) and P-wave velocity (*V_p_*) of lime-stabilized basalt fiber-reinforced loess. Reinforced loess samples with different proportions of basalt fiber and lime were subjected to 0, 1, 5, and 10 freeze–thaw cycles, and their UCS and *V_p_* were subsequently measured. The test results showed that the addition of basalt fiber and lime to loess could enhance strength and improve resistance against freeze–thaw damage, and the freeze–thaw damage of reinforced loess decreases with the increase of basalt fiber content and length. A relationship between UCS and *V_p_* of the reinforced samples was obtained for the same number of freeze–thaw cycles, and this relationship exhibited linear characteristics. The fitting results indicate that the *V_p_* can be used to estimate the UCS after freeze–thaw damage. The research results not only have important practical significance in the application of basalt fiber in geotechnical engineering but also provide a reference for the non-destructive testing of the strength of loess after freeze–thaw cycles.

## 1. Introduction

Loess is broadly distributed worldwide and covers approximately 10% of all land surfaces [1,2]. In contrast to ordinary sand and clay, loess is a unique soil in which the tubular pores are well developed. Due to the high latitude of the loess distribution area, it is often affected by freeze–thaw cycles. When loess is subjected to freeze–thaw treatment, the strength and deformation characteristics change significantly due to expansion and contraction in volume of pore water [3]. Consequently, engineering structures with slopes and embankments in areas with seasonally frozen loess are in danger of damage due to large cracks, settlements, and slope deformations [4,5,6]. Therefore, the prevention of freeze–thaw damage has important engineering implications.

Many scholars have studied different fibers in soil curing and have obtained significant results [7,8]. Akbulut, et al. [9] researched strengthening the mechanical characteristics of clay with fibers obtained from solid waste such as waste tires. Shahbazi, et al. [10] considered the reinforcement effect of fibers and slag on expansive soil. Ramesh, et al. [11] found that the mechanical properties of fiber-reinforced black cotton soil with lime added are appreciably better than black cotton soil alone with fibers. Currently, many researchers are studying different types of additive fibers to effectively mitigate the adverse effects of freeze–thaw processes on engineering materials. Zaimoglu [12] showed that a fiber-reinforced sample exhibited more ductility than a non-reinforced one. The most notable effect was observed on 0.75% polypropylene fiber-reinforced samples. Ghazavi and Roustaie [13] demonstrated that adding polypropylene fiber did reduce the freeze–thaw damage on soil strength. Roustaei, et al. [14] found that the effect of freeze-thaw cycles on cohesion decreased with the increase of fiber content in the reinforced soil. Güllü and Khudir [15] studied the effects of freeze–thaw cycles on the strength of soil reinforced by different fibers. In addition, composite materials composed of biomaterials and natural fibers have great application potential in soil reinforcement due to their high mechanical strength, low toxicity, good barrier properties, and environmentally-friendly processing [16,17,18].

Previous studies have demonstrated that applying different fibers as reinforcements has a notable impact on the static, dynamic, and thermal behaviors of soils [19,20,21]. However, only a few studies have used basalt fibers to control freeze–thaw damage. Basalt fiber has unique characteristics, such as good ductility, high strength, and high-temperature resistance, and appears to have less environmental impact than glass fiber and petroleum chemical fiber [22,23]. Xu, et al. [24] and Jiang, et al. [25] found that basalt fibers were significantly more effective in reinforcing sandy soils than glass fibers and polyvinyl alcohol fiber. Gao, et al. [26] found that basalt fiber’s inclusion enhanced the cohesive strength of clay, and its reinforcement effect increased with the increasing fiber content. Orakoglu and Liu [27] also found that the reduction in strength caused by freeze–thaw damage with a higher basalt fiber content is relatively minor. Davar, et al. [28] detected that basalt fiber’s addition could make up for the strength reduction of the asphalt mixtures at low temperatures. Boz, et al. [29] pointed out that adding lime can affect the reinforcement effect of the fiber on clay. Some scholars made preliminary investigations into the reinforcement mechanism of basalt fibers on loess but have not considered the effect of freeze–thaw cycles that loess is often subjected to [30,31]. Table 1 summarizes the research on the application of basalt fibers for soil reinforcement in geotechnical engineering. According to results obtained from the above studies, there is insufficient information on the characterization of lime-stabilized soil strengthened by basalt fiber. In addition, there is no comparative study on the freeze–thaw effect of lime-stabilized soil reinforced by basalt fiber at different fiber lengths and lime contents. 

The propagation speed of waves in rocks and soils is closely related to their mechanical behaviors [32,33]. Measuring the change in P-wave velocity (*V_p_*) to evaluate freeze–thaw damage is a non-destructive technique [34,35]. Takarli and Prince [36] found that freeze–thaw damage can cause a decrease in *V_p_* of granite and pointed out that this was caused by the formation of new micro-cracks due to freeze–thaw damage. Walbert, et al. [37] found a similar phenomenon in the study of limestone. However, few scholars have studied the effect of different freeze–thaw cycles on the wave velocity of soil reinforced by fiber and lime.

In embankment filling or foundation treatment, it is essential to maintain the strength of reinforced and stabilized soil. The primary purpose of this study was to quantitatively investigate the influence of the addition of basalt fiber and lime on the strength and internal structure of loess under different freeze–thaw cycles. *V_p_* is often used to characterize the integrity of the internal structure of rock and soil. In the research of this paper, an unconfined compression test and bender element test were conducted to measure the UCS and *V_p_* of the reinforced loess. The effects of fiber content and length and lime content on the UCS and *V_p_* after freeze–thaw cycles were measured, and the relationship between the *V_p_* and UCS was analyzed. The research results not only have important practical significance in applications of this new reinforcement additive, basalt fiber, in geotechnical engineering but also provide a reference for the non-destructive testing of the strength of loess after freeze–thaw cycles.

## 2. Materials

The test materials were loess, basalt fiber, and lime. The loess was collected from an open pit construction field (from depths of 5–6 m) near the city of Taiyuan in North China. This area belongs to North China’s seasonally frozen regions. The average winter temperatures in this area range from −8 °C to +4 °C, and the lowest temperature recorded in winter was −26 °C. To ensure the stability of the open pit slope, it is planned to excavate the loess at the top of the slope and fill it at the foot of the slope to reduce the slope ratio. It is proposed to add basalt fiber to the loess to enhance its strength. Considering the well-developed tubular pores inside the loess, lime will be added to enhance the reinforcement effect of basalt fibers. To provide a basis for the design of the new slope reinforcement and stability analysis, it was necessary to quantitatively study the reinforcement effect of basalt fiber and lime on loess.

**Figure 1:**
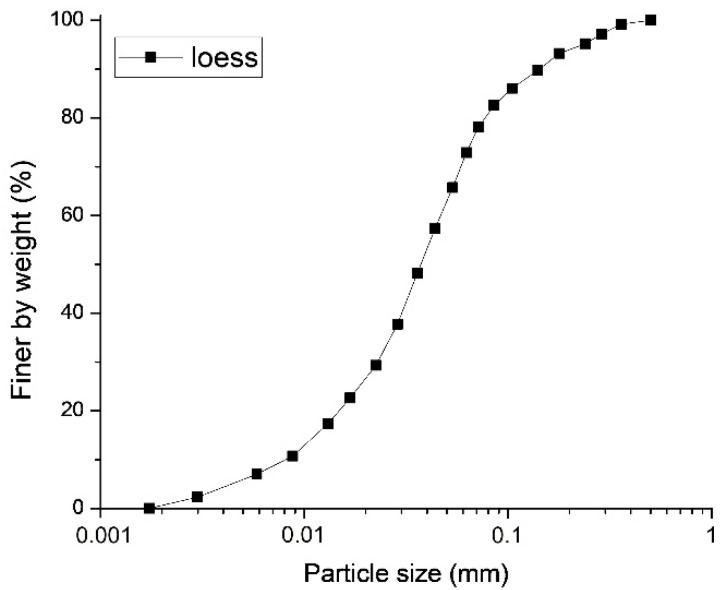
Particle size gradation curve of loess.

The loess samples were brought for experimental analyses into a laboratory, where grain size gradation curves were plotted, and mechanical parameters were tested according to the Chinese national standard, Standard for geotechnical testing method (GB/T 50123—2019), as presented in Table 2. It must be noted that before the specimen preparation, particles of size greater than 2 mm were removed to maintain consistency among the specimens. The particle size of the loess samples was tested three times using a Microtrac S3500 analyzer (Montgomeryville, PA, USA), as shown in Figure 1. It can be observed from the grain size analysis that the loess samples could be classified as silty soil. 

The basalt fibers used in the study were obtained from Yibin Basalt Fiber Technology Company in Sichuan Province, China. Basalt fiber is comprised of oxides such as silica, alumina, calcium oxide, magnesium oxide, iron oxide, and titanium dioxide [38,39]. The basalt fiber material parameters were obtained from the manufacturer and are listed in Table 3 [40].

## 3. Testing Procedure

### 3.1. Specimen Preparation

**Figure 2:**
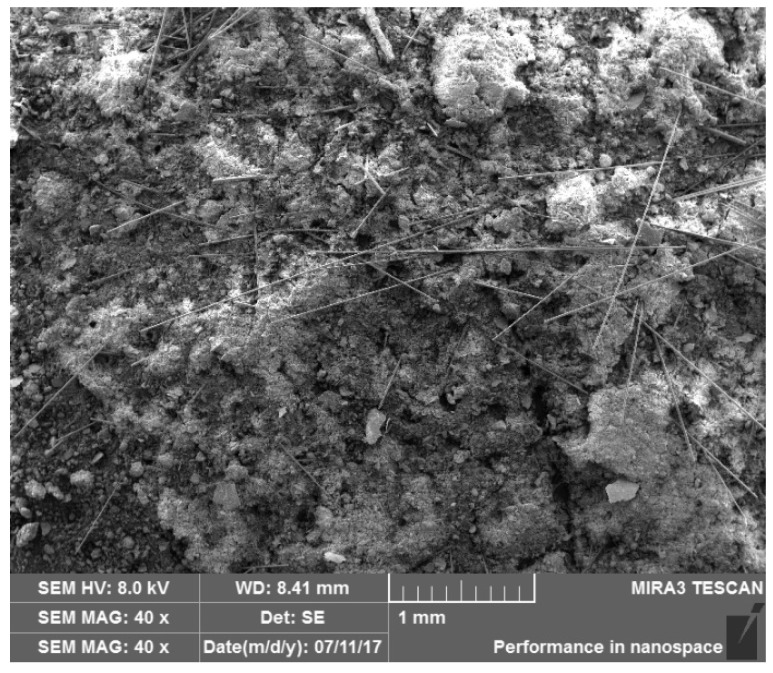
Scanning electron micrograph of the reinforced loess.

First, samples of loess mixtures containing 6 mm and 12 mm basalt fibers were prepared. The dry loess was mixed evenly with different contents of basalt fibers while avoiding flocculation. The mixture was then stabilized with 1% and 3% lime, respectively, and an appropriate amount of water was added to the air-dried loess to achieve an optimal moisture content of 13%. The mixture was then placed in a sealed plastic bag for 24 h to achieve an even water distribution. Cylindrical specimens were then prepared, with lengths and diameters of 80 mm and 39.1 mm, respectively. The fiber-reinforced loess samples were cured for 28 days at a relative humidity of 95 ± 2% and a temperature of 24 ± 1 °C. The electron micrograph of the fiber-reinforced loess sample is shown in Figure 2. It was observed that the basalt fibers in the loess are interwoven and distributed to form a network structure. Lime fills the pores between the loess particles and ensures adherence between particles, particles and fibers, and fibers and fibers.

After curing, the samples were subjected to freeze–thaw processes using a refrigerator. To prevent the specimens from absorbing moisture during the thawing process, each specimen was covered by a homemade box made of foam and plastic cloth during the freeze–thaw process. In the freezing phase, the samples were placed inside a −15 °C refrigerator with the optimum water content and frozen for 12 h. The samples were then removed from the refrigerator, air-conditioned in a 24 °C humid chamber, and thawed for 12 h. This entire process was the basis of one freeze–thaw cycle.

### 3.2. Test Method

The unconfined compression test and bender element test were carried out on the reinforced loess samples using the test scheme provided in Table 4.

**Figure 3:**
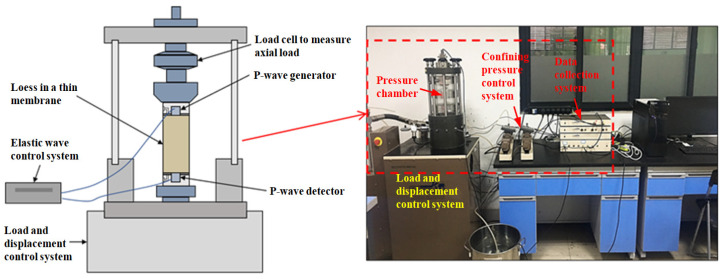
GDS triaxial test system.

The tests were conducted using GDS triaxial test equipment (Hook, Hampshire, UK). The equipment is shown in Figure 3. The lime-stabilized fiber-reinforced loess samples were placed into the test machine, and then the *V_p_* was measured by the bender element test. The unconfined compression test was then performed at a 0.8 mm/min compression rate.

As shown in Figure 3, the P-wave was generated by an additional piezoelectric device in the top cap and was detected in the pedestal by a similar device through the loess specimen [41,42,43]. The principle is to generate P-waves by changing the wiring structure [44,45]. The installation procedure comprises one element (P-wave generator) installed on the top cap while the other element (P-wave detector) is installed on the pedestal. The test requires the two elements to be inserted into the soil specimen separately. An external controller serves as a power supply and an amplifier. In this study, a single sine wave with a period of 0.5 ms and an amplitude of input voltage of 14 V was selected as the excitation signal. To ensure the test conditions’ consistency, the same excitation signal was used in all the bender element tests. The calculation formula of *V_p_* is shown below [46,47,48,49]:

*V_p_* = *L*_0_/Δ*t* (1)

where *L*_0_ is the effective propagation distance, that is, the height of the specimen minus twice the height of the thin piezo-ceramic plate in the bender element, and Δ*t* is the propagation time of the P-wave, which is the time difference between the peak of the excitation signal and the peak of the arrival signal.

## 4. Results Analysis and Discussion

### 4.1. Axial Stress–Strain Curves

**Figure 4:**
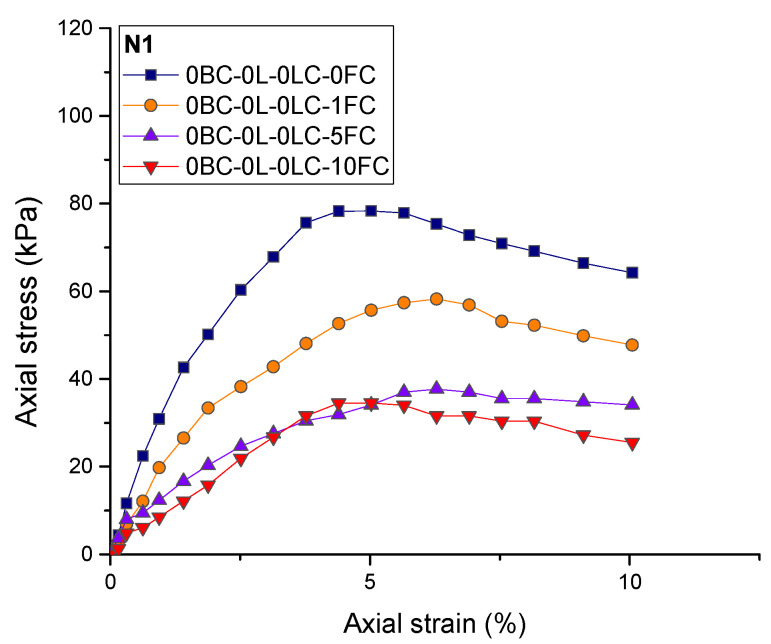
Stress–strain curves of loess without basalt fiber and lime reinforcement.

The effects of basalt fiber content, length, and lime content on the UCS of reinforced loess were investigated. The nature of the sample is indicated by the following symbols and numbers. For example, the 0.5BC-6L-1LC-1FC denotes a specimen with a basalt fiber content of 0.5%, fiber length of 6 mm, lime content of 1%, and is subjected to 1 freeze–thaw cycle. Figure 4 shows the axial stress–strain curves of the loess without fiber and lime reinforcement under freeze–thaw treatment. As observed in Figure 4, the UCS of the loess samples without the addition of fiber and lime is only 78 kPa, and after 10 freeze–thaw cycles, it becomes 34 kPa.

**Figure 5:**
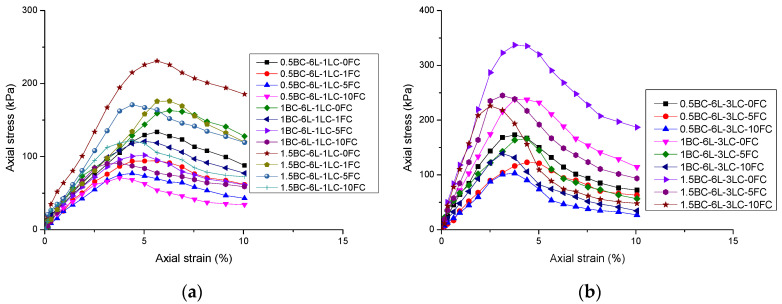

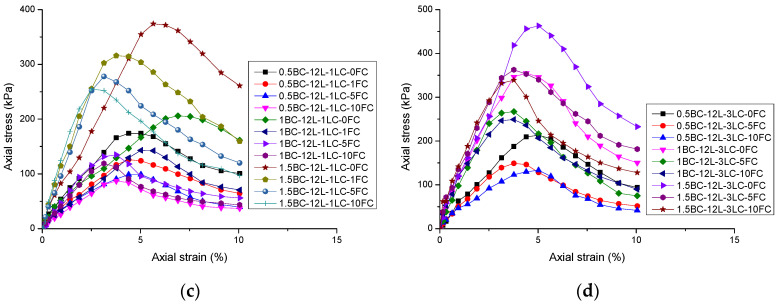
Stress–strain curves of reinforced loess samples with different basalt fiber and lime contents: (**a**) BC = 0.5, 1 and 1.5%, L = 6 mm, LC = 1%; (**b**) BC = 0.5, 1 and 1.5%, L = 6 mm, LC = 3%; (**c**) BC = 0.5, 1 and 1.5%, L = 12 mm, LC = 1%; and (**d**) BC = 0.5, 1 and 1.5%, L = 12 mm, LC = 3%.

Figure 5 shows the axial stress–strain curves of the reinforced loess with different fiber contents, fiber lengths, lime contents, and freeze–thaw cycles. The phenomenon of strain-softening appears in the stress–strain relationship of reinforced loess. Despite the addition of fiber and lime, as the number of freeze–thaw cycles increased, the UCS of all samples decreased. At 0 and 10 freeze–thaw cycles, the average damage axial strain corresponding to the UCS was 5.1% and 3.5%, respectively. This indicates that as the number of freeze–thaw cycles increases, the corresponding axial strain decreases when the axial stress peak appears. This is because when the loess sample is in the freezing phase, the volume of pore water expands during the freezing process. As a result, the soil particles become separated, enlarging the inter-particle gap, and microcracks are generated in the test specimens. However, during the subsequent thawing phase, the increased voids and microcracks do not fully recover to their original state before the next freezing [50,51].

Figure 4 and Figure 5 show that with the increase in freeze–thaw cycles, there is a lack of regularity in the relationship between axial strain and UCS, which may be caused by the complexity and heterogenous influence of freeze–thaw cycles on the internal structure of loess specimens. The freeze–thaw cycle expands the specimen volume and loosens the particle structure, resulting in a complex stress transmission process and strain increase process under compression load [52].

**Figure 6:**
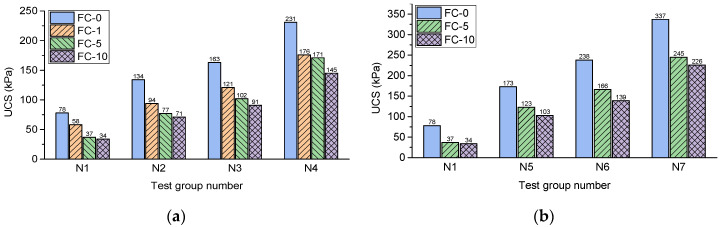

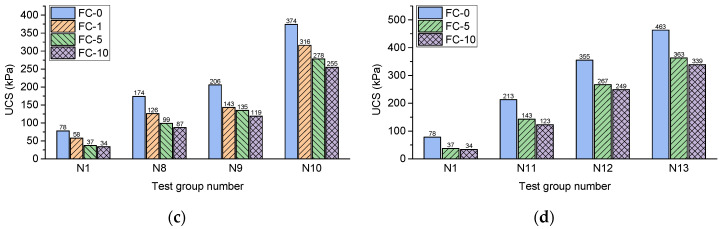
Effects of basalt fiber and lime on UCS under different freeze–thaw cycles: (**a**) L = 6 mm and LC = 1%; (**b**) L = 6 mm and LC = of 3%; (**c**) L = 12 mm and LC = 1%; and (**d**) L = 12 mm and LC = 3%.

Figure 6 shows the UCS values of the experimental groups. It is observed that the experimental group with the strongest reinforcing effect is 1.5BC-12L-3LC (N13). The UCS values of this experimental group were 463 kPa and 339 kPa at the 0 and 10 freeze–thaw cycles, respectively, which corresponded to increases 493% and 897%, respectively, compared with loess without reinforcement. This shows that the basalt fiber and lime act as curing additives for loess and possess sound strength-enhancing effects. Fiber reinforcement is a physical process that combines the fiber’s tensile strength with the soil’s compressive strength by using the friction force between the fiber and the soil. The interaction of lime as an inorganic binder with soil belongs to the domain of chemical enhancement. This interaction makes the fibers and the soil particles better connected.

### 4.2. Effects of Basalt Fiber Content and Length

**Figure 7:**
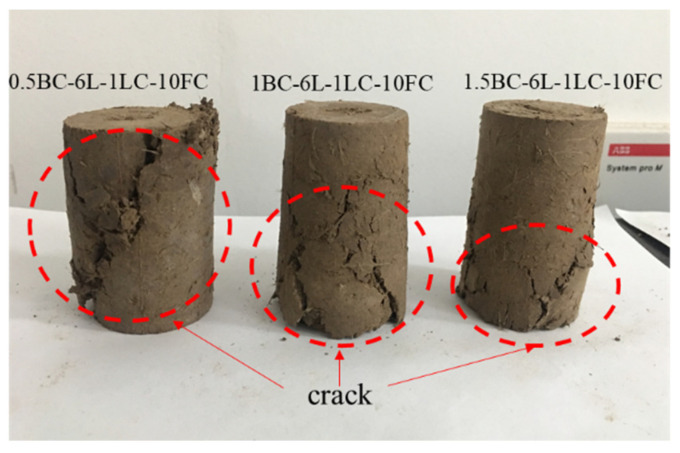
Failure forms of samples with different basalt fiber contents.

From Figure 6a it can be seen that the UCS increases with the increase in basalt fiber content. For example, under the conditions of 0 freeze–thaw cycle and 1% lime content, with the content of basalt fiber with 6 mm length increasing from 0.5% to 1.5%, the UCS increased from 134 kPa to 231 kPa. This shows that the basalt fiber content has a considerable effect on the strength of the loess sample. The uniaxial compression failure witnessed in the experiment also indicated that the destruction forms of the samples with the three fiber contents differ, as shown in Figure 7. The samples with 1% and 1.5% basalt fiber content after loading showed visible cracks in the lower part of the samples. However, the cracks run throughout the sample with 0.5% basalt fiber content. Zheng, Zhang, Liu, Yang and Yang [40] analyzed the mechanical behaviors at the interface of different tailing particles and fibers through the scanning electron microscopy test. There is adhesion and friction between fibers and particles, and the interaction between fibers is mainly due to the occlusion between fibers. This is also the main microscopic form of fiber-reinforced loess. Since the fiber distribution is uniform during the overall reinforcement, the fiber always prevents the cracks during generation and expansion. The expansion direction of cracks changes several times, increasing failure strain. The study of Lenoir, Preteseille and Ricordel [19] also shows that reinforcement actively stabilizes sandy clay materials. The natural matrix is tightly attached to fibers, which can prevent the occurrence of microcracks.

**Figure 8:**
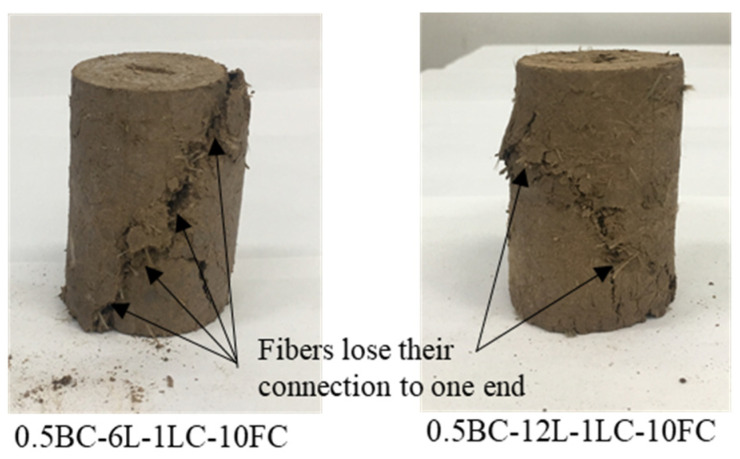
Failure forms of samples with 6 mm and 12 mm fiber length.

Similarly, the increase in basalt fiber length also increases the UCS. By comparing the two experimental groups in Figure 6a,c, the UCS values of the 12 mm basalt fiber with the same content and freeze–thaw cycle were higher than those in the 6 mm basalt fiber experimental group. For example, comparing the 0 to 10 freeze–thaw cycles, the UCS of the 6 mm fiber-reinforced samples with 1.5% fiber contents ranged from 231 kPa to 145 kPa, whereas that of the 12 mm samples ranged from 374 kPa to 255 kPa. The longer the fiber, the more extensive the range of particles that a single fiber can contact. The failure of reinforced loess often occurs after a large deformation has occurred. When more significant deformation occurs, the longer the fiber, the more likely it is to maintain contact with the particles. Meanwhile, by comparing the failure forms of the two fiber length samples in Figure 8, it was found that the crack width of the 6 mm basalt fiber was significantly wider than that of the 12 mm basalt fiber. The longer fibers could still connect the two ends to provide a bonding force for the compression cracks, while the shorter fibers could only have one connection. This also indicates that the 12 mm fiber can better enhance the strength of loess than the 6 mm fiber. Chaduvula, et al. [53] found that as the length and content of fibers in clay increased, the crack size and intensity both decreased, which was similar to the results in this study.

Within the scope of the experimental conditions in this article, the strength of basalt fiber reinforced loess increases with the increase of the basalt fiber content and length. However, when the basalt fiber content and length are further increased, the strength of the reinforced loess may not improve. The research results of Gao, Hu, Xu, Fu, Xiang and Yang [26] show that when the basalt fiber content and length are 0.25% and 12 mm, respectively, the UCS of the basalt fiber reinforced clay reaches a maximum. Further increases in the content and length of basalt fiber will decrease the strength of the reinforced clay.

During the process of freeze–thaw treatments, the volume of pore water in loess expands, which reduces the contact area between loess particles and fibers, and the particle-fiber structure becomes loose. Therefore, the freeze–thaw damage to fiber-reinforced loess increases with the number of freeze–thaw cycles. To examine the effect of basalt fiber length and content on the freeze–thaw damage, we defined the damage value D as follows:

D = (UCS_FC-0_ − UCS_FC-n_)/UCS_FC-0_ × 100% (2)

where n represents the number of freeze–thaw cycles.

**Figure 9:**
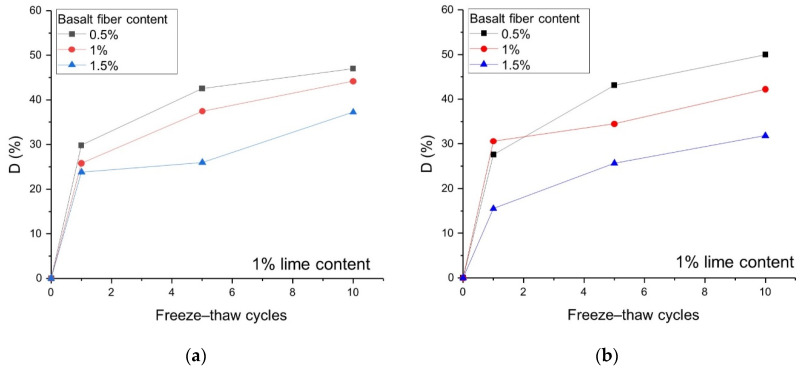
Effects of fiber content and length on the freeze–thaw damage D with 1% lime content: (**a**) L = 6 mm and LC = 1%; (**b**) L = 12 mm and LC = 1%.

It is observed in Figure 9a that after 10 freeze–thaw cycles, the D values of 6 mm length basalt fiber with 0.5, 1, and 1.5% contents are 47, 44, and 37%, respectively. Meanwhile, the D values of 12 mm length fiber with the same contents (Figure 9b) are 50, 42, and 31%, respectively. This clearly shows that increasing the fiber content can reduce the freeze–thaw damage. Simultaneously, the calculation results also show that the 12 mm fiber has a better effect in reducing the freeze–thaw damage than the 6 mm fiber.

**Figure 10:**
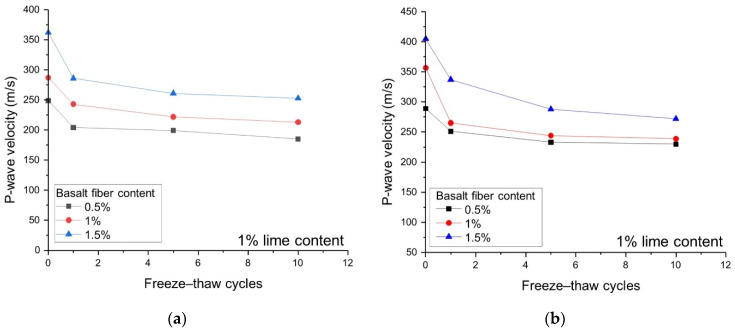
Effects of fiber content and length on the wave velocity with 1% lime content: (**a**) L = 6 mm and LC = 1%; (**b**) L = 12 mm and LC = 1%.

Figure 10 shows the effect on *V_p_* of fiber content, length, and freeze–thaw cycles. As in the samples with 6 mm fiber length (Figure 10a), the *V_p_* ranges of the 0.5% and 1.5% fiber contents are 185 m/s to 249 m/s and 253 m/s to 362 m/s, respectively. As for the 12 mm fiber length (Figure 10b), the *V_p_* values of the 0.5% and 1.5% fiber content range from 230 m/s to 289 m/s and 272 m/s to 405 m/s, respectively. The *V_p_* value is positively correlated with fiber content and length. This is because the increase in the content and the length of basalt fiber increases the continuity within the sample, and the propagation velocity of P-wave in solids is greater than that in liquids and gases, thereby increasing the *V_p_* value of reinforced loess.

### 4.3. Effects of Lime Content

In Figure 6, with a 6 mm fiber length and 0 freeze–thaw cycles, the UCS values of the samples with 3% lime content and 0.5, 1, and 1.5% fiber content increased by 39 kPa, 75 kPa, and 106 kPa, respectively, compared with UCS values of the 1% lime content samples. The experimental results prove that the UCS values of the samples with more lime are higher than those with less lime. Simultaneously, the stress peak can be reached at a more minor axial strain, as shown in Figure 5. This is because the pozzolanic reaction between the silica and alumina in the loess particles, the pore water, and the added lime will lead to several curable substances [5,50,54], which further enhances the strength. 

**Figure 11:**
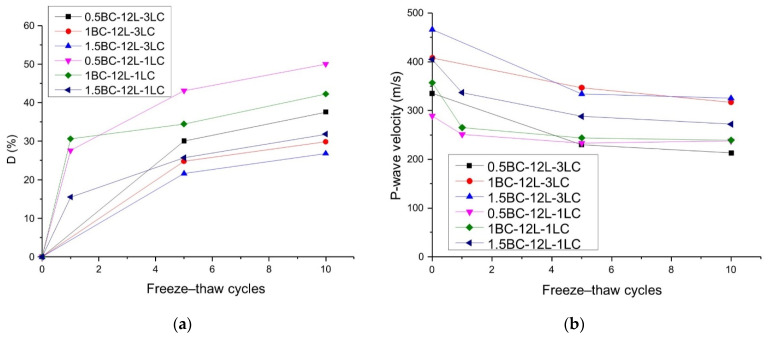
Effects of lime content and freeze–thaw cycles on freeze–thaw damage and wave velocity with 12 mm fiber length: (**a**) freeze–thaw damage D; and (**b**) P-wave velocity.

Figure 11 shows the D and *V_p_* values of the 1% and 3% lime stabilized samples. From Figure 11a, it can be concluded that the increase in lime content is beneficial in reducing the freeze–thaw damage. Similarly, for samples having the same basalt fiber content and length, the *V_p_* value of 3% lime content sample is greater than that of the sample with 1% lime content, indicating that the solidified matter generated by lime has high particle adhesion.

### 4.4. Relationship between UCS and V_p_

Figure 10 and Figure 11b present the bender element test results of different experimental groups. *V_p_* decreases with the increasing freeze–thaw cycles. For example, in the 0.5BC-6L-1LC group with 0 to 10 freeze–thaw cycles, *V_p_* drops from 249 m/s to 185 m/s. This is because as the number of freeze–thaw cycles increases, the volume of pores and microcracks in the wave propagation zone of the reinforced sample increase, causing the path length of the wave propagation to be extended. Yarbaşı, et al. [55] have also reached similar conclusions.

**Figure 12:**
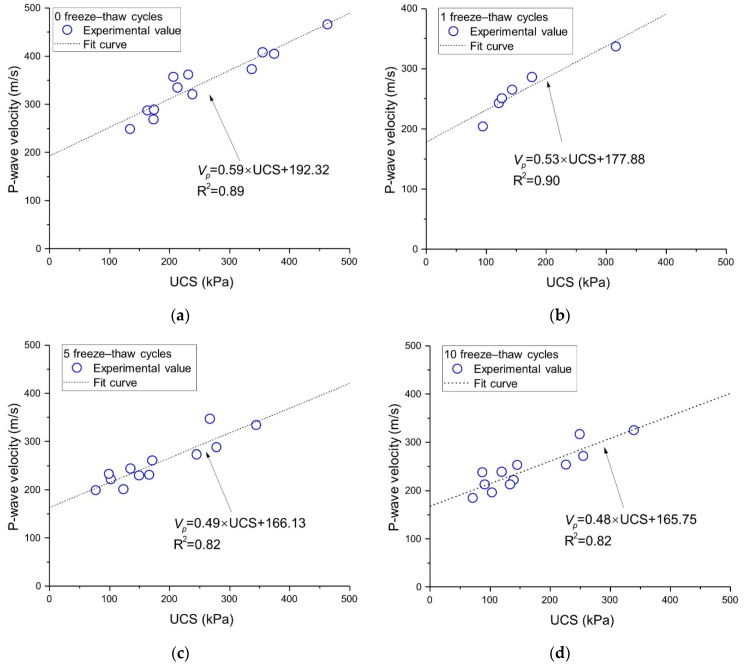
Relationship between *V_p_* and UCS: (**a**) FC = 0, (**b**) FC = 1, (**c**) FC = 5, and (**d**) FC = 10.

Wave-velocity testing technology is a non-destructive testing method [56,57]. To predict UCS after freeze–thaw damage, the UCS and *V_p_* of basalt fiber-reinforced loess after different freeze–thaw cycles were plotted. The correlations between the UCS and *V_p_* for samples reinforced by lime and fiber under 0, 1, 5, and 10 freeze–thaw cycles are shown in Figure 12. The UCS–*V_p_* relationship can be fitted with a linear curve, which is similar to the conclusion arrived at by Boz and Sezer [58] and Choobbasti, et al. [59]. As can be observed from Figure 12, with the increase in freeze–thaw cycles, the slope of the fitted curve changes without obvious regularity, but the intercept shows a decreasing trend, from 192.32 kPa to 165.75 kPa. The goodness of fit (R^2^) of all linear fitting formulas is greater than 0.8, indicating that the UCS value of the fiber-reinforced loess under different freeze–thaw cycles can be estimated approximately by measuring the *V_p_*.

## 5. Conclusions

As an environmentally-friendly material, basalt fiber is prevalent in soil reinforcement for geotechnical construction. However, few studies have reported on the reinforcement effect of basalt fibers on the freeze–thaw damage in loess. In this study, an extensive experimental investigation was conducted to evaluate the improvement in the freeze–thaw performance of lime-stabilized basalt fiber-reinforced loess with different basalt fiber contents and lengths and lime contents. The effects of fiber content and length and lime content on the UCS and *V_p_* after being subjected to freeze–thaw cycles were measured. The test results showed that adding basalt fiber and lime to loess could enhance strength and improve resistance against freeze–thaw damage. In the basalt fiber content range of 0.5% to 1.5%, the loess sample with the larger fiber content had a higher UCS and *V_p_* after the freeze–thaw treatment, and the 12 mm length fibers enhanced the strength of the reinforced loess samples more than the 6 mm length fibers. The relationship between the UCS and *V_p_* of the reinforced loess samples with the same freeze–thaw cycles exhibited linear characteristics, so the UCS of the frozen–thawed loess could be approximated by non-destructive testing of the *V_p_*. The findings of this study indicated that the application of basalt fiber reinforcement and lime stabilization could be an effective treatment for significantly improving the freeze–thaw damage performance of loess.

## Figures and Tables

**Table 1:** Research on the application of basalt fibers for soil reinforcement.

Reinforcement Material	Type of Soil	Test Content	Author and Year
Basalt fiber	Cement soil	Splitting tensile strength	Wang et al., 2020 [20]
	Clay	Static and dynamic tensile strength, freeze-thaw damage	Gao et al., 2020 [21]
	Kaolinite	Uniaxial and triaxial compressive strength	Wang et al., 2020 [22]
	Silty clay	Uniaxial compressive strength	Tao et al., 2022 [23]
	Sand	Simple shear strength	Xu et al., 2021 [24]
	Uranium tailing	Compressive strength, radon exhalation rate	Jiang et al., 2022 [25]
	Clay	Uniaxial compressive strength	Gao et al., 2015 [26]
	Clay	Triaxial compressive strength, freeze–thaw damage	Orakoglu and Liu 2017 [27]
	Asphalt	Tensile strength, fatigue damage	Davar et al., 2017 [28]
	Clay	Uniaxial compressive strength	Boz et al., 2018 [29]
	Loess	Triaxial compressive strength	Xu et al., 2021 [30,31]

**Table 2:** Physical parameters of loess.

Parameter	Value
Specific gravity	2.71
Maximum dry density (g/cm^3^)	1.71
Cohesion (kPa)	22.3
Internal friction angle (°)	21.5
Liquid limit (%)	33.9
Plastic limit (%)	20.9
Moisture content range of sample (%)	12–15

**Table 3:** Performance parameters s of basalt fibers.

Elastic Modulus (GPa)	Tensile Strength (MPa)	Fracture Strength (MPa)	Density (g/cm^3^)
89	2650	3200	2.7

**Table 4:** Test scheme.

Loess Sample No.	Basalt Fiber Content (%)	Fiber Length (mm)	Lime Content (%)	Freeze–Thaw Cycles			
N1	0	0	0	0	1	5	10
N2	0.5	6	1	0	1	5	10
N3	1	6	1	0	1	5	10
N4	1.5	6	1	0	1	5	10
N5	0.5	6	3	0	-	5	10
N6	1	6	3	0	-	5	10
N7	1.5	6	3	0	-	5	10
N8	0.5	12	1	0	1	5	10
N9	1	12	1	0	1	5	10
N10	1.5	12	1	0	1	5	10
N11	0.5	12	3	0	-	5	10
N12	1	12	3	0	-	5	10
N13	1.5	12	3	0	-	5	10

## Data Availability

The data presented in this study are available on request from the corresponding author. The data are not publicly available due to privacy.
